# Correction: Evaluating the Accuracy of Diffusion MRI Models in White Matter

**DOI:** 10.1371/journal.pone.0139150

**Published:** 2015-09-22

**Authors:** Ariel Rokem, Jason D. Yeatman, Franco Pestilli, Kendrick N. Kay, Aviv Mezer, Stefan van der Walt, Brian A. Wandell

There is an error in Equation 3. Please see the correct Equation 3 here.

$$\text{S}\left(\theta ,\text{b}\right)={\text{ β}}_{o}e^{-bD}+\overset{F}{\underset{i=1}{\sum }}\beta_{i}e^{-b\theta^{t}Q_{i}\theta }$$
