# Supplementary figures and images for: Evaluating the Accuracy of Diffusion MRI Models in White Matter
Source: PLoS One. 2015 Apr 16;10(4):e0123272. doi: 10.1371/journal.pone.0123272 (PMC4400066; doi:10.1371/journal.pone.0123272)

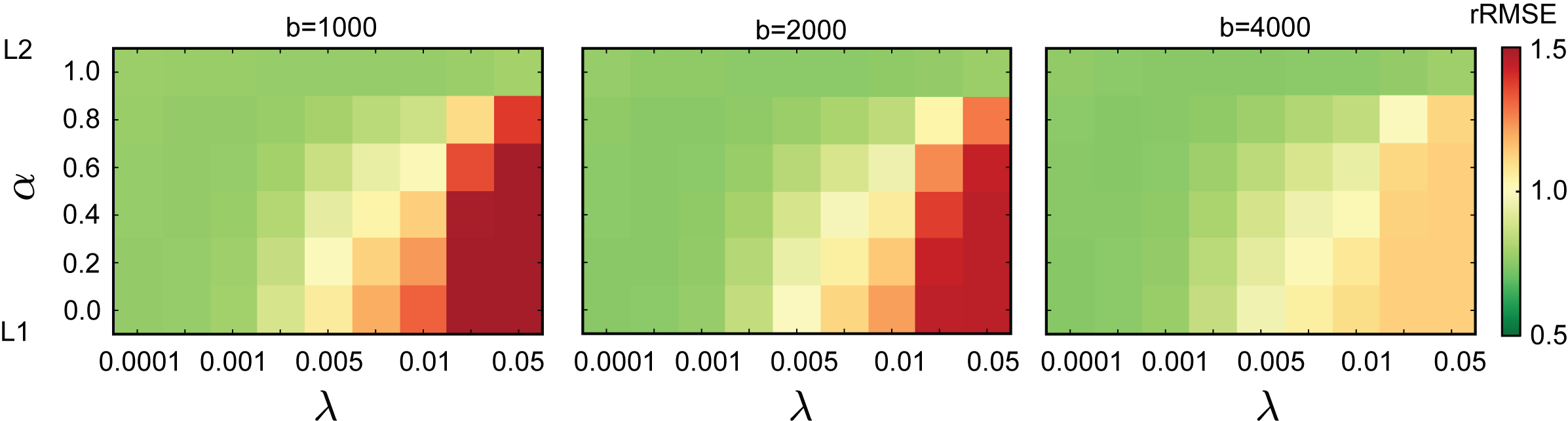

Supplement: S1 Fig — We used regularization and cross-validation to (a) prefer solutions that minimize the number of fascicles and (b) prevent over-fitting. To find the appropriate setting of the regularization parameters λ and α, we used a cross-validation approach. The SFM was fit on one set of data for a range of λ and α values. For each combination the SFM was fit to one data set and the prediction error was calculated using the other data set. We choose λ and α that minimize the median rRMSE across white matter voxels. We explore the effects of regularization and the trade-off of different sets of constraints on the accuracy of the fit. The best setting of these parameters is to a relatively low degree of regularization (λ = 0.0005) and relatively L1-weighted constraint (α = 0.2). These are the parameters used in all the SFM model fits. (TIF) [file pone.0123272.s001.tif]
